# Enhancement of Therapeutic mRNA Translation in Cellular Stress Conditions

**DOI:** 10.3390/ijms27114663

**Published:** 2026-05-22

**Authors:** Edyta Trepkowska-Mejer

**Affiliations:** Laboratory of Experimental Medicine, Faculty of Medicine, Medical University of Warsaw, Żwirki i Wigury 61, 02-091 Warsaw, Poland; edyta.trepkowska@wum.edu.pl

**Keywords:** mRNA therapeutics, cellular stress, alternative translation, mRNA design, innovative therapy

## Abstract

This review summarizes mechanisms regulating mRNA translation under cellular stress and highlights design strategies to improve translation efficiency and stability in the gene therapy of human diseases. mRNA-based therapeutics are emerging as a versatile gene therapy platform enabling transient and controllable expression of therapeutic proteins for the treatment of cancer, genetic disorders, and inflammatory diseases. The efficacy of mRNA-based gene therapy is strongly influenced by sequence design, chemical modifications, and structural features. Evidence shows that rational mRNA engineering can significantly enhance translation efficiency even under stress conditions that impair canonical protein synthesis, as observed in many pathological states. Cellular stress activates regulatory pathways that suppress global translation; however, optimized mRNA constructs can partially bypass these inhibitory mechanisms, enabling sustained protein expression. By improving mRNA stability and resistance to stress-responsive translational control, robust therapeutic protein production can be achieved even in challenging cellular environments. This article was prepared as a narrative review focused on translational regulation mechanisms relevant to therapeutic mRNA design under cellular stress conditions. Literature was collected from PubMed, Google Scholar, and Web of Science using keywords including “mRNA therapeutics,” “cellular stress,” “translation regulation,” “UTR engineering,” and “cap-independent translation.” Studies published mainly between 2010 and 2025 were considered. Original articles and reviews related to stress-responsive translation and therapeutic mRNA optimization were included, while studies outside the scope of translational control and mRNA engineering were excluded. Priority was given to recent and mechanistically relevant publications.

## 1. Introduction

Over the past decade, owing to technological innovations and research investments, mRNA has emerged as a promising therapeutic tool in the field of vaccine development and replenishment of missing proteins. Rapid advances in RNA biology, delivery technologies, and chemical engineering have transformed RNA molecules into a major class of therapeutic agents. RNA-based drugs now represent a diverse and rapidly expanding field that extends far beyond prophylactic vaccination. This class includes small interfering RNAs (siRNAs), antisense oligonucleotides (ASOs), microRNA (miRNA) mimics and inhibitors, aptamers, and messenger RNA (mRNA)-based therapeutics encoding proteins or antibodies. These modalities act at different levels of gene expression regulation, ranging from transcriptional and post-transcriptional control to direct protein replacement, and are being actively developed or already approved for a wide spectrum of diseases, including oncology, rare genetic disorders, cardiovascular and metabolic diseases, neurodegeneration, and infectious diseases. Recent comprehensive analyses of RNA therapeutics underscore this expanding clinical landscape and highlight the increasing importance of RNA-based drug repurposing and platform adaptability in modern pharmacology [1]. As this approach allows for fast and efficient optimization of mRNA sequences, these medicines have the potential to revolutionize both the prevention and treatment of diseases. The use of mRNA has several advantages over killed and live attenuated viruses used in traditional vaccines [2]. First, safety: because an mRNA is a non-infectious molecule that does not integrate into genomic DNA, there is no risk of potential infection or insertional mutagenesis [3]. Within this broader context, therapeutic mRNA has emerged as a particularly versatile platform for transient protein expression. Its clinical relevance became especially evident during the rapid development of mRNA vaccines, but its potential extends well beyond prophylaxis. mRNA-based therapeutics are being actively explored for protein replacement therapies, cancer immunotherapy, regenerative medicine, and the delivery of therapeutic antibodies and cytokines [1]. Furthermore, mRNA is degraded by physiological cellular processes; its half-life can be regulated by the structure of the coding sequence of the mRNA itself, as well as by delivery methods [4]. The inherent immunogenicity of mRNA can be reduced to further improve its safety profile [5]. Second, efficacy: various modifications stabilize the molecule and ensure high translation. In vivo delivery can be achieved by formulating mRNA in the carrier molecules, enabling rapid uptake and mRNA expression in the cytoplasm [6]. Third, mRNA vaccines can be manufactured rapidly, inexpensively, and scalably [7].

The mRNA sequence consists of five key elements. First, a protective molecule at the 5′ end called the cap, which consists of an atypical nucleoside, 7-methylguanosine, joined by a 5′,5′-triphosphate linkage to the 5′ end of the mRNA chain, which protects the mRNA from premature degradation by 5′ exonuclease [8]. The cap is involved in many other biological functions including maturation, transport, and translation initiation. The 5′ untranslated region (5′UTR) plays a crucial role in the regulation of translation processes, as it has an impact on stability, localization, and translation initiation by interacting with ribosomes and initiation factors [9,10,11]. Immediately after the 5′UTR, a protein-coding sequence is located, and the 3′ untranslated region (3′UTR) is placed behind the stop codon. The 3′UTR is typically longer than the 5′UTR; it influences mRNA stability, translation efficiency, and cellular localization as it interacts with RNA binding proteins and microRNAs [12,13]. The last element of the mRNA sequence is a 3′ poly-(A) tail that contributes to the mRNA translational status and decay as a result of interaction with poly(A)-binding proteins and deadenylases [14,15].

The central design consideration in mRNA medicine is its nucleotide sequence, which affects the structure, function, and stability of mRNA. The interplay between selected codon triplets, RNA secondary and tertiary structures, and nucleotide modification is crucial for the fulfillment of the target therapeutic profile [16]. For all organisms and even tissues, codon biases exist; more frequently used ones correlate with the abundance of tRNAs [17]. Mammalian mRNA sequences are generally GC-rich, with a strong emphasis on C as the third nucleotide in the codon. This phenomenon leads to increased mRNA stability and secondary structure formation [18]. Next, the spatial structure of the mRNA can positively or negatively influence the entire translation process. mRNA forms complex secondary structures as a result of intramolecular base pairing, which enables the binding of protein complexes and influences storage longevity [4,16].

In addition to optimizing the protein-coding sequence, appropriate selection of UTRs modulates translation towards increasing or decreasing translation efficiency. Unstructured 5′UTRs, in general, ensure better translation initiation [19] suggesting the simplest design of these elements; however, rapid initiation leads to faster mRNA decay, affecting total protein expression [20]. The selection of the appropriate cap analog is also important as the interaction of initiation factors with the 7-methylguanosine cap is one of the rate-limiting steps in the entire translation process [21]. The poly-(A) tail is another mRNA design that influences stability and translation efficacy.

Designing therapeutic mRNA is therefore a complex optimization problem in which multiple, often competing, parameters must be balanced. Maximizing protein expression from delivered mRNA molecules depends on its half-life in the target tissue and translational potential, which is related to the number of ribosomes scanning and transiting mRNA [22]. However, ribosome overload leads to translation mRNA decay, lowering total protein yield and eliciting toxic effects; thus, appropriate ribosome loading is highly needed [23]. Other factors include ease and consistency of manufacturing, as well as regulation of mRNA shelf life, both unformulated and in its delivery vehicle [24]. Because mRNA molecules are inherently unstable and susceptible to degradation, extending mRNA stability is one of the key requirements for this group of therapeutics.

Despite major advances in mRNA therapeutics, most optimization strategies have focused on improving stability and translation under normal cellular conditions. However, therapeutic mRNAs are often delivered into diseased tissues characterized by hypoxia, inflammation, oxidative stress, or viral infection, where canonical cap-dependent translation is impaired. Under these conditions, cells activate stress-responsive translation programs that selectively maintain the synthesis of survival-related proteins. Consequently, conventional mRNA optimization may not ensure efficient protein expression in stressed cells. Translation modulation can be achieved by engineering mRNA sequences adapted to the cell state. As changes in translation are a key feature of many diseases such as cancer, viral infections, and myocardial hypertrophy [25], designing mRNA therapeutics whose translation involves mechanisms induced by cellular stress may lead to an expression advantage when canonical translation is inhibited [26].

This review introduces the concept of “stress-aware mRNA design,” which involves incorporating stress-adaptive translational mechanisms into therapeutic mRNA engineering. Unlike conventional approaches, stress-aware design aims to exploit alternative translation pathways, stress-responsive UTRs, and RNA structural elements that remain active during cellular stress. While previous reviews have discussed either mRNA therapeutics or stress-induced translational control separately, here, we integrate these fields and focus on how stress-associated regulatory mechanisms can be rationally applied to improve therapeutic mRNA performance in pathological environments.

## 2. Canonical Mode of mRNA Translation

To understand translational adaptation to cellular stress, it is necessary to briefly outline the core features of canonical mRNA translation under physiological conditions, with particular emphasis on steps that are most sensitive to regulatory control. Eukaryotic protein synthesis proceeds through three main phases: initiation, elongation, and termination coupled with ribosome recycling [26,27]. Among these, translation initiation represents the primary regulatory and rate-limiting step, integrating signals from multiple initiation factors, ribosomal subunits, mRNA, and tRNAs.

In canonical cap-dependent translation initiation, the 40S ribosomal subunit is recruited to the mRNA to form the 43S pre-initiation complex (43S PIC), which subsequently scans the 5′ untranslated region (5′UTR) in a 5′ → 3′ direction until start codon recognition occurs. This process depends on coordinated actions of eukaryotic initiation factors (eIFs), including the eIF2–GTP–Met-tRNAi ternary complex and the eIF4F cap-binding complex, which is assembled by eIF4E, eIF4G, and the RNA helicase eIF4A. ATP-dependent helicase activity of eIF4A facilitates resolution of secondary structures within the 5′UTR, thereby enabling efficient ribosomal scanning and start codon access [28,29].

**Figure 1:**
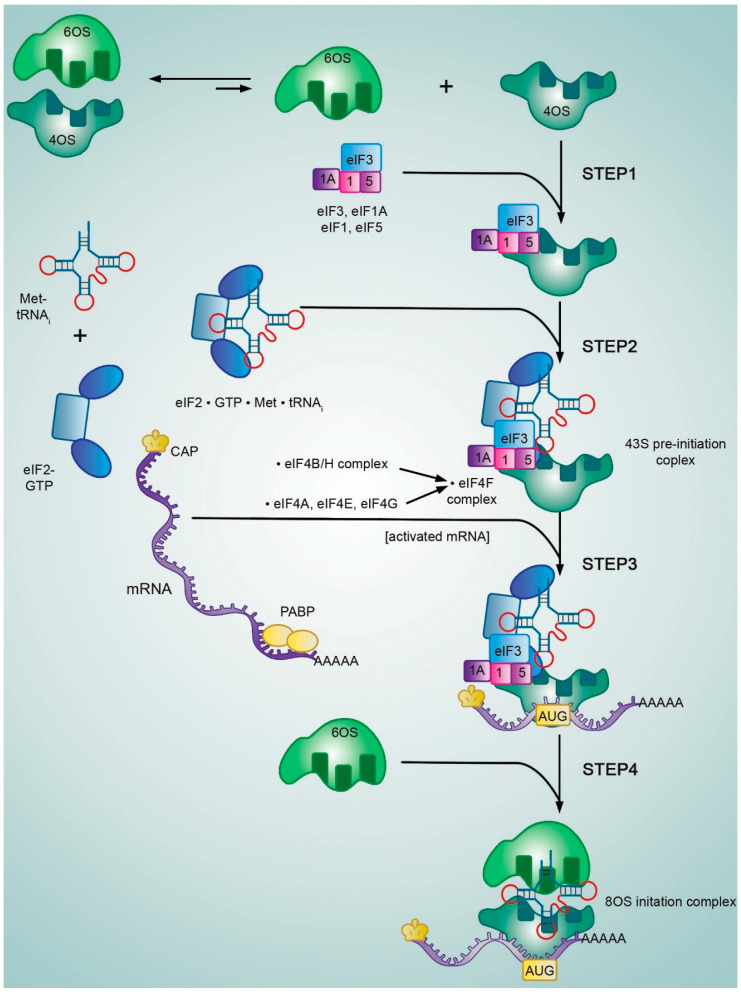
Schematic representation of canonical mode of translation initiation. At the beginning, a small ribosome subunit (40S) binds with eukaryotic initiation factors eIF3, eIF1, eIF1A and eIF5. Also, eukaryotic translation initiation factor 2 (eIF2) and GTP associate with methionyl-transfer RNA, leading to ternary complex formation. Next, 43S pre-initiation complex formation occurs as a result of ternary complex binding to the 40S ribosome subunit. The cap-binding complex, consisting of eIF4A, eIF4E and eIF4G, binds to the 5′ cap of the mRNA; eIF4G also associates with the poly(A)-binding protein (PABP). The activated mRNA, complexed with initiation factors, binds to the 43S pre-initiation complex and scanning of the mRNA occurs to find the AUG start codon. In the last step, GTP is hydrolyzed by eIF2, which enables the dissociation of the initiation factors from the 48S complex, thereby allowing binding of the large 60S ribosomal subunit, resulting in the formation of the 80S ribosome, ready for translation elongation and protein synthesis.

Start codon recognition typically occurs at an AUG codon embedded within an optimal Kozak consensus sequence [30,31], triggering GTP hydrolysis events mediated by initiation factors such as eIF2 and eIF5B, and promoting joining of the 60S ribosomal subunit to form the translationally competent 80S ribosome. Notably, initiation factor recycling (e.g., eIF2B-mediated nucleotide exchange) and cap-dependent recruitment via eIF4E represent key regulatory checkpoints that determine translational output. Figure 1 shows a schematic representation of the canonical mode of translation initiation. Importantly, these canonical initiation steps constitute major nodes of vulnerability to cellular stress pathways described in subsequent sections. Stress-induced phosphorylation of eIF2α, modulation of cap-dependent translation through eIF4F inhibition, and alterations in ribosome scanning efficiency collectively reprogram global and selective mRNA translation.

## 3. mRNA Translation Initiation in Cellular Stress and Disease

The inhospitable environmental conditions under which multicellular life evolves and persists require specialized mechanisms for the efficient repair of molecular damage. This pressure allows all cells to adapt to adverse environmental conditions. Upon exposure to physiological intra- and extracellular stress stimuli, eukaryotic cells activate an adaptive pathway called the integrated stress response (ISR), which reprograms cellular metabolism and focuses on maintaining homeostasis [32]. The ISR is activated under most types of stress stimuli, including endoplasmic reticulum (ER) stress (which induces the unfolded protein response [33]), hypoxia [34], nutrition deprivation [35], heat shock [36], viral infection [37], oxidative stress [38], UV irradiation [39], and proteasome inhibition [40].

## 4. Cellular Pathways Involved in Stress Responses

### 4.1. Stress-Induced eIF2α Phosphorylation and Disease Development

Upon activation of the integrated stress response (ISR) by diverse cellular stressors, phosphorylation of eIF2α at Ser51 leads to a rapid attenuation of global cap-dependent translation and a coordinated reprogramming of the cellular proteome. This translational control mechanism reduces overall protein synthesis to conserve resources and limit proteotoxic stress, while selectively enabling the expression of stress-adaptive transcripts that support cell survival or, under unresolved stress, promote apoptosis [33,34]. Importantly, ISR signaling is not solely determined by kinase activity but is dynamically balanced by dedicated phosphatase complexes. The PP1 catalytic subunit, in association with regulatory proteins GADD34 (PPP1R15A) and CReP (PPP1R15B), mediates dephosphorylation of eIF2α and thereby controls the temporal duration of translational repression [41]. GADD34 is transcriptionally induced during stress and forms a negative feedback loop that restores translation during recovery, whereas CReP provides constitutive basal dephosphorylation activity. The balance between kinase-driven phosphorylation and PP1c-mediated dephosphorylation determines the effective “translational window” during which selective mRNA translation can occur, which is particularly relevant for therapeutic mRNA expression under stress conditions. Selective translation under ISR conditions is strongly influenced by 5′UTR architecture. A major regulatory mechanism involves upstream open reading frames (uORFs), which enable stress-dependent reinitiation of translation. Phosphorylation of eIF2α reduces ternary complex availability, thereby altering reinitiation dynamics and enabling preferential translation of transcripts such as ATF4, which encode key stress-response regulators [27]. In parallel, internal ribosome entry sites (IRESs) facilitate cap-independent translation initiation when eIF4E-dependent cap recognition is compromised, enabling continued synthesis of selected stress-response proteins, including BiP (HSPA5) and Apaf-1 [42].

**Figure 2:**
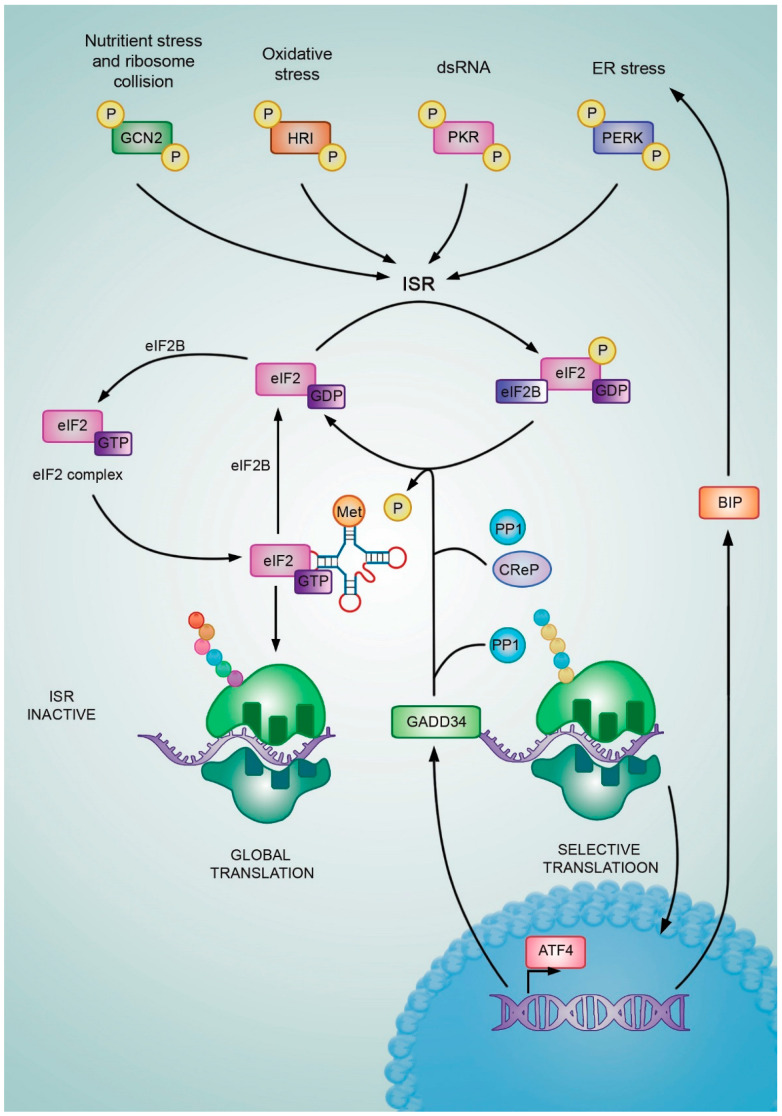
eIF2α phosphorylation and ISR program. Upon various different stimuli, kinases GCN2 (amino acid starvation and UV radiation), HRI (oxidative stress and heat shock), PKR (viral infection and double-stranded RNA) and PERK (ER stress, hypoxia and proteostasis) phosphorylate eIF2 at Ser-51 of its α-subunit, preventing GDP to GTP exchange by eIF2B; thus, the ternary complex cannot be regenerated. eIF2α phosphorylation leads to global cap-dependent translation inhibition and promotes selective, stress-responsive mRNA translation.

Additional layers of regulation arise from RNA secondary structures and RNA-binding proteins that remodel 5′UTR accessibility under stress. Stress-induced RNA-binding proteins such as HuR and TIA-1 can selectively enhance or repress translation of structured mRNAs, contributing to stress-specific proteome remodeling [43]. Collectively, these mechanisms enable a highly selective translational program that preserves cellular adaptability while suppressing bulk protein synthesis. Figure 2 illustrates eIF2α phosphorylation and the activation of the integrated stress response (ISR) program.

### 4.2. Regulation of eIF4E Activity Under Cellular Stress

eIF4E plays an essential role in regulating the expression of proliferation and survival proteins; therefore, its action is strictly controlled. To date, several cellular mechanisms have been described in the context of eIF4E activity, including mammalian target of rapamycin (mTOR) signaling through eIF4E-binding proteins (4E-BPs), phosphorylation of eIF4E by Mnk, and stimulation of eIF4E transcription by Myc.

#### The mTOR Pathway

Cap-dependent translation initiation via eIF4E is regulated by competitive interactions with eIF4E-binding proteins (4E-BPs), which control assembly of the eIF4F complex. eIF4E promotes translation through binding of eIF4G, whereas hypophosphorylated 4E-BPs sequester eIF4E and prevent eIF4F formation, thereby repressing translation initiation [44,45]. A key regulatory layer involves hierarchical, multisite phosphorylation of 4E-BP1 by mechanistic target of rapamycin complex 1 (mTORC1). 4E-BP1 is phosphorylated in a stepwise manner at multiple residues, including T37/T46 (priming sites), followed by S65 and T70 (inhibitory release sites), which progressively reduce its affinity for eIF4E [46]. In parallel with this graded control, mTORC1 regulates translational output through phosphorylation of downstream effectors, including S6 kinases (S6Ks), which promote ribosomal protein synthesis, translation factor production, and inhibition of eukaryotic elongation factor 2 kinase (eEF2K), thereby enhancing translational elongation efficiency under growth-promoting conditions [42].

The upstream regulator of mTORC1 activity is class I phosphoinositide 3-kinase (PI3K), a key signaling enzyme activated by secreted ligands including hormones (such as insulin), growth factors, cytokines, and chemokines [47]. Another signaling pathway controlled by mTORC1 involves 5′-adenosine monophosphate-activated protein kinase (AMPK), which is essential for cellular metabolism and growth [48].

Beyond mTORC1, mTORC2 also contributes to translational regulation, particularly in cancer and stress-adapted states. Although traditionally associated with cytoskeletal regulation and AKT activation, mTORC2 indirectly influences translation through sustained AKT signaling, which promotes mTORC1 activity and enhances cap-dependent translation in tumor cells. In addition, emerging evidence suggests that mTORC2 may support translational reprogramming under stress conditions by maintaining AKT-dependent survival signaling, thereby sustaining selective mRNA translation in environments where mTORC1 activity is fluctuating or partially inhibited [49].

**Figure 3:**
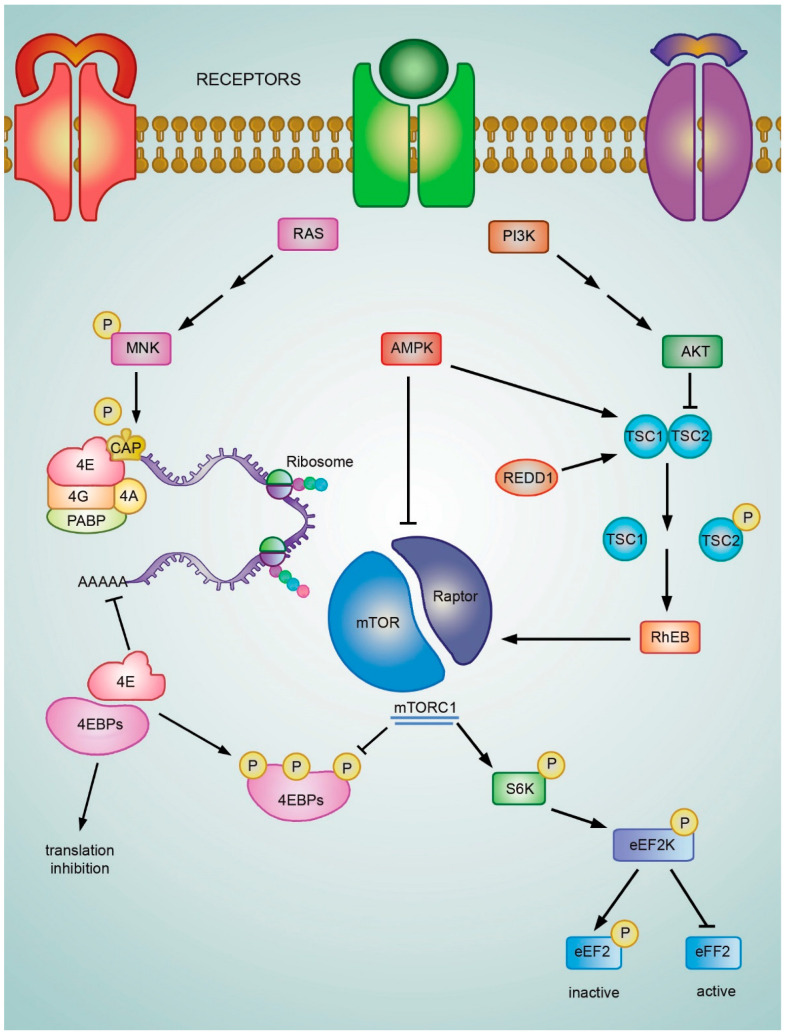
Distinct mechanisms regulating eIF4E activity and cap-dependent translation during cellular stress. Extracellular signal-regulated kinase (ERK) activation stimulates MAPK-interacting kinases (MNKs), resulting in phosphorylation of eIF4E, which modulates selective mRNA translation and stress-responsive gene expression. Independently, growth factor signaling activates the PI3K/AKT pathway, leading to inhibition of the TSC1/TSC2 complex and activation of mTORC1. Activated mTORC1 phosphorylates 4E-BPs, promoting eIF4E release and cap-dependent translation initiation, while also activating S6 kinases (S6Ks) to support translational elongation through regulation of eukaryotic elongation factor 2 (eEF2). In contrast, stress-induced pathways such as the integrated stress response suppress global cap-dependent translation, thereby functionally distinguishing growth-associated translational activation from stress-induced translational reprogramming.

mRNAs most sensitive to eIF4E availability upon mTOR belong to specific classes defined by distinct 5′UTR features. Canonical examples include 5′ terminal oligopyrimidine (5′TOP) and TOP-like mRNAs encoding ribosomal proteins and translation factors, whose translation is acutely suppressed when mTOR–4E-BP signaling is inactive [50]. In addition, mRNAs with long, GC-rich, highly structured 5′UTRs, such as MYC, CCND1, VEGFA, and ODC1, exhibit strong dependence on eIF4E and are preferentially downregulated when cap-dependent initiation is limited [11]. These findings underscore that mTOR signaling selectively controls translation of defined mRNA subsets rather than uniformly regulating protein synthesis. In Figure 3, eIF4E activity regulation in the stress response is presented.

## 5. mRNA-Specific Regulation

### 5.1. Trans-Acting Factors Regulate mRNA Fate in Cellular Stress Response

Trans-acting factors are proteins and RNAs that recognize specific elements in mRNA molecules and facilitate rapid changes in gene expression in response to cellular stress. Diverse mechanisms are involved in the action of RNA-binding proteins and non-coding regulatory RNAs such as microRNAs and tRNA-derived RNAs [51,52,53,54].

#### 5.1.1. RNA-Binding Proteins (RBPs)

RBPs play an important role in the mRNA life cycle [55]. This is implemented by interacting with sequence elements in the 5′ and 3′ untranslated regions and relies on the recruitment of mRNAs to the ribosome and regulation of protein synthesis or, alternatively, repression of mRNA translation by regulating mRNA instability and decay [56]. Under oxidative stress, there is a strong association between transcripts that are translationally repressed and those associated with the RNA-binding protein PUF3p, a well-known regulator of proteins targeted to mitochondria. PUF-3p-responsive elements are short UGUA-containing motifs in the 3′UTR that confer stress-dependent regulation of mRNA stability and translation; avoiding these elements can therefore allow therapeutic mRNAs to escape oxidative stress-induced translational shutdown [57]. In turn, upon DNA damage, RBP HuR/ELAV1 reduces its binding to mRNA partners MDM2 and BAX, which enhances cell survival [58]. Given that HuR recognizes AU-rich elements (AREs) in 3′UTR, insertion of these motifs into therapeutic mRNA may enhance translation compared to non-ARE mRNAs [59].

#### 5.1.2. MicroRNAs

MicroRNAs are short RNAs consisting of around 22 nucleotides that modulate the translational potential of their target mRNA [60]. Mature miRNAs bind to the 3′UTR via the seed region. The degree of complementarity determines cleavage (exact matching) or translation repression (partial matching). Multiple forms of stress, including hypoxia, DNA damage, ER stress, and inflammatory signaling, alter miRNA transcription, processing, and target engagement [53]. For therapeutic mRNAs delivered into stressed tissues, these dynamics present an opportunity: rather than attempting to override stress-induced translational repression globally, mRNA constructs can be designed to cooperate with endogenous miRNA stress responses. One of the most consistently observed features of cellular stress is the selective downregulation of specific miRNAs. In cancer and ischemic tissues, hypoxia can reduce global miRNA biogenesis through inhibition of Drosha and Dicer, leading to broad attenuation of miRNA-mediated repression [61]. From a therapeutic design perspective, inclusion of target sites for stress-downregulated miRNAs in the 3′UTR of a therapeutic mRNA can create a conditional expression switch. Under stress, loss of the miRNA relieves repression, resulting in relative translational enhancement [62]. Although miRNAs are most often associated with repression, several studies have demonstrated that miRNAs can activate translation in specific cellular states. For example, miR-369-3 directs translational activation of TNF-α mRNA in quiescent cells by recruiting AGO2 and FXR1 to AU-rich elements [63].

#### 5.1.3. tRFs and tiRNAs

Other non-protein-coding RNAs are tRNA fragments that serve as precursors for subsequent cleaving agents, generating two classes of functional RNA fragments: 17–26-nucleotide long tRNA-derived RNA fragments (tRFs) and tRNA-derived stress-induced RNAs (tiRNAs) [64]. It has been previously reported that ANG cleaves tRNAs nonspecifically during cellular stress, such as UV irradiation, heat shock, arsenite treatment, nutrition deficiency, hypoxia, and hypothermia [65,66]. While many tiRNAs repress global cap-dependent translation, specific tRFs modulate translation in a sequence-, context-, and RBP-dependent manner, including interactions with Argonaute proteins and stress-response RBPs [65,66]. Mechanistic studies have demonstrated that tRNA-derived fragments can selectively repress translation of mRNAs containing specific UTR motifs by displacing RNA-binding proteins such as YBX1 under stress conditions, suggesting that incorporation of tRF- or tiRNA-responsive elements into therapeutic mRNA UTRs could be exploited to modulate translation during stress adaptation [67].

### 5.2. Specific Cis-Acting Features of mRNA Control the Rate and Mode of Translation During Stress

Selective translation during cellular stress is governed by cis-acting regulatory elements embedded within mRNA untranslated regions and proximal coding sequences, including the 5′ cap, 5′UTR, and 3′UTR. These elements act in a coordinated and context-dependent manner to fine-tune translation efficiency, ribosome recruitment, and transcript stability [68]. Although their mechanistic roles are well characterized in endogenous transcripts, their applicability to engineered therapeutic mRNAs remains variable and highly dependent on cellular context and stress intensity.

#### 5.2.1. Stress-Induced mRNA Modification

Post-transcriptional RNA modifications, particularly *N*6-methyladenosine (m^6^A) and 2′-*O*-methylation, modulate translation by altering RNA structure and recruiting specific reader proteins such as YTH domain-containing factors. In endogenous systems, m^6^A has been shown to promote cap-independent translation under conditions of impaired cap-dependent initiation and similar effects have been observed primarily in reporter assays and cell-based models. However, the extent to which strategically introduced m^6^A sites can reliably enhance therapeutic mRNA translation in vivo under complex stress conditions remains incompletely defined [69,70].

#### 5.2.2. Upstream Open Reading Frames (uORFs)

Upstream open reading frames (uORFs) represent a central mechanism of stress-dependent translational reprogramming by modulating ribosome scanning and reinitiation in an eIF2α-dependent manner [71]. Their role is well established in endogenous stress-responsive transcripts such as ATF4, CHOP, and BiP, where they enable selective translation during integrated stress-response activation [72,73,74,75,76]. Synthetic incorporation of uORFs has demonstrated controllable effects in reporter and cell-based systems, yet quantitative predictability across diverse stress intensities and tissues remains an open challenge.

#### 5.2.3. 5′Terminal Oligopyrimidines (5′TOP) Motifs

5′ terminal oligopyrimidine (5′TOP) motifs constitute a major regulatory class of mTOR-sensitive transcripts encoding ribosomal and translational machinery components [77]. Importantly, translation is not strictly binary under stress conditions, as partial mTOR inhibition allows residual cap-dependent translation of specific transcripts [78,79]. This graded behavior provides a conceptual basis for engineering therapeutic mRNAs responsive to intermediate mTOR activity states, although robust in vivo validation remains limited.

#### 5.2.4. Translation Initiator of Short 5′UTR (TISU)

Translation Initiator of Short 5′UTR (TISU) elements enable efficient initiation in transcripts with minimal 5′UTR complexity and reduced dependence on scanning-based regulation [80,81]. Although their function is well characterized in endogenous gene expression, their behavior under pathological stress conditions and in therapeutic mRNA contexts has not been systematically explored.

#### 5.2.5. Internal Ribosome Entry Site (IRES)

Internal ribosome entry sites (IRESs) enable cap-independent recruitment of ribosomes and are widely utilized in viral systems and select cellular transcripts involved in stress responses [82]. Approximately 15% of the total cell mRNA can be translated via the IRES mechanism; however, only about 100 transcripts contain IRES elements, suggesting that IRES-containing mRNAs are usually translated in a cap-dependent mechanism and switch to an IRES-dependent mechanism under stress [83]. By incorporating viral or cellular IRES upstream of the therapeutic mRNA ORF, the translation continues even in stress conditions such as hypoxia, viral infection or oxidative stress [84]. Certain IRES elements (e.g., VEGF, HIF-1α, and c-Myc) are naturally more active under specific stresses; thus, usage of stress-responsive cellular IRES allows for control of therapeutic mRNA translation selectively in stressed cells [85]. However, their activity in mammalian cells is highly context-dependent and remains controversial in terms of mechanistic consistency across experimental systems [86]. Consequently, while IRES elements provide a conceptually attractive strategy for stress-resilient translation, their performance in engineered therapeutic mRNAs is difficult to generalize and requires careful empirical validation.

#### 5.2.6. Cap-Independent Translation Enhancers (CITEs)

Cap-independent translation enhancers (CITEs), originally characterized in viral RNAs, facilitate initiation by recruiting translation machinery independently of the 5′ cap structure [87]. 5′CITE-like structures can bind eIF4F in a cap-independent manner, providing a moderate dependence on the 5′cap and making their translation, at least partially, resistant to stress-induced inactivation [88]. Viral or synthetic CITE-like RNA structure utilization in therapeutic mRNAs enables recruitment of initiation factors (e.g., eIF4G, eIF4A) directly to the mRNA, ensuring sustained translation despite global repression of cap-dependent initiation [89]. Although these elements are well established in plant viral systems, their adaptation to mammalian translation remains largely experimental and has not yet been validated as a robust or broadly applicable strategy for therapeutic mRNA design [90].

#### 5.2.7. Alternative Cap-Dependent Mechanism in Stress Response

Finally, non-canonical cap-dependent translation mediated by eIF3 subunits, particularly eIF3D, represents an emerging layer of translational control that becomes prominent under metabolic stress conditions [91,92], suggesting a potential role in stress-adaptive translation. However, its exploitation in engineered mRNA systems remains largely theoretical and requires further mechanistic and in vivo validation.

Collectively, cis-regulatory elements define a modular but highly context-sensitive framework for translational control. While their mechanistic foundations are well established in endogenous RNA biology, their translation into predictive design rules for therapeutic mRNAs is still limited by incomplete quantitative understanding, context dependency, and insufficient in vivo validation under physiologically relevant stress conditions. Bridging this gap will require systematic comparative studies across stress models to establish reliable design principles for stress-responsive mRNA therapeutics. Table 1 presents stress-responsive translational regulatory mechanisms and their validation status in mRNA engineering.

## 6. Preclinical and Translational Evidence for Engineered mRNA Design Strategies

Although multiple cis-regulatory and structural RNA engineering strategies have been extensively characterized at the mechanistic level, only a subset has been validated in preclinical in vivo models, while most engineered applications remain restricted to in vitro or reporter-based systems. Importantly, none of the described strategies has yet achieved clinical validation as a therapeutic mRNA design principle. An in vivo validation layer exists for uORF-mediated translational control; endogenous uORF-dependent regulation, particularly within the integrated stress-response (ISR) axis involving ATF4, has been demonstrated in a mouse model, where eIF2α phosphorylation drives adaptive translational reprogramming in tissues such as the liver and pancreas [102]. These findings establish uORFs as a physiologically validated stress-response mechanism in vivo; however, synthetic uORF engineering for therapeutic mRNA remains largely limited to reporter assays and cell-based systems, with only partial validation in animal models. Similarly, mTOR–5′TOP signaling represents a robust in vivo-validated translational control pathway. Pharmacological inhibition of mTORC1 using agents such as rapamycin (Rapamycin) in mouse models results in suppression of 5′TOP-driven translation of ribosomal protein mRNAs, confirming the physiological relevance of this regulatory axis in vivo [103].

While this provides a strong mechanistic basis for engineering stress-responsive mRNAs, direct demonstration of 5′TOP-based therapeutic mRNA performance in disease models remains absent. In contrast, m^6^A-mediated translational enhancement shows mixed evidence strength. In vivo studies in mice have demonstrated that m^6^A dynamics regulate mRNA translation under physiological and stress-related conditions, particularly via reader-dependent mechanisms [69]. However, engineered insertion of m^6^A motifs into therapeutic mRNAs has so far been validated primarily in vitro or in cell-based reporter systems, with no robust disease-model validation confirming predictable translational enhancement in vivo.

Internal ribosome entry site (IRES)-mediated translation has moderate in vivo support, primarily derived from viral RNA systems. Viral IRES elements, such as those from EMCV, have been shown to sustain translation in mammalian cells and in vivo under conditions where cap-dependent initiation is impaired [86]. Nevertheless, the efficiency of IRES-driven translation is highly context-dependent, and synthetic application in therapeutic mRNA remains poorly standardized in animal models. Cap-independent translation enhancers (CITEs) are largely restricted to in vitro and plant viral systems, with only limited and indirect evidence of activity in mammalian experimental settings [104]. No convincing in vivo mammalian disease model has yet demonstrated robust CITE-mediated enhancement of therapeutic mRNA expression. Finally, non-canonical cap-dependent translation mediated by eIF3d has emerging but limited in vivo support, primarily from cancer models. Mouse tumor studies have shown that metabolic stress can activate eIF3-dependent selective translation programs, contributing to adaptive protein synthesis under nutrient and oxygen limitation [105]. However, its exploitation as a programmable element in therapeutic mRNA design remains entirely preclinical and conceptual.

Collectively, the available evidence demonstrates a clear stratification of translational maturity. uORF- and mTOR–5′TOP-dependent mechanisms are strongly supported by in vivo physiological models, whereas m^6^A, IRES, and eIF3d exhibit partial or context-dependent in vivo validation. In contrast, CITE-based systems remain largely restricted to in vitro proof-of-concept studies. Crucially, no cis-regulatory engineering strategy has yet reached clinical validation, highlighting a significant translational gap between mechanistic RNA biology and therapeutic mRNA design.

## 7. Coupling of Stress-Responsive Translation with RNA Processing and Innate Immune Pathways

Efficient therapeutic mRNA translation depends not only on RNA design but also on the performance of delivery systems, which determine cytosolic access to the translational machinery. Lipid nanoparticles (LNPs), currently the most clinically advanced platform, as used in Comirnaty and Spikevax, rely on endocytosis and endosomal escape, a major rate-limiting step for productive mRNA expression. Cellular stress states significantly influence these processes. Oxidative and inflammatory stress can alter endosomal membrane composition and trafficking dynamics, potentially modifying escape efficiency, whereas hypoxia can impair vesicular transport and membrane fluidity, reducing cytosolic delivery. These effects are particularly relevant in pathological tissues such as tumors and inflamed microenvironments, where stress signaling is chronically activated [106].

Polymeric carriers and lipid–polymer hybrid systems are similarly affected by stress-dependent changes in endosomal maturation, acidification, and lysosomal degradation pathways, which collectively influence mRNA release efficiency [107]. Extracellular vesicle-based systems also exhibit stress-sensitive uptake and trafficking behavior due to altered receptor expression and membrane remodeling in recipient cells, although in vivo quantitative data remain limited [108]. Importantly, cellular stress simultaneously impacts translation capacity. Integrated stress response activation and mTOR inhibition can suppress global protein synthesis even when cytosolic mRNA delivery is successful, creating a functional bottleneck between delivery and translation. This highlights the need to consider delivery and translational control as an integrated system rather than independent processes [109].

Emerging stress-responsive delivery systems aim to exploit pathological microenvironments by using triggers such as pH changes, reactive oxygen species, or inflammation-associated enzymes to enhance site-specific mRNA release. When combined with stress-adaptive mRNA designs, these approaches may improve protein expression selectively under disease conditions, although most evidence currently remains preclinical [110,111].

To better integrate the discussion of stress-responsive translational design with other determinants of therapeutic mRNA performance, it is important to emphasize the role of RNA quality attributes and innate immune sensing pathways. In addition to sequence-encoded translational regulation, factors such as dsRNA impurities generated during in vitro transcription, cap structure integrity, poly-(A) tail heterogeneity, and nucleotide modifications critically influence both mRNA stability and translational efficiency. These features directly modulate the activation of innate immune sensors, including PKR, OAS/RNase L, and RIG-I-like receptors, which can induce global translational arrest through phosphorylation of eIF2α and RNA degradation pathways [112,113,114]. Consequently, innate immune activation represents a central bottleneck that can override otherwise optimized mRNA design, particularly under stress conditions where antiviral and inflammatory signaling is already elevated.

Importantly, stress-responsive translational control mechanisms intersect with these immune pathways at multiple levels. For example, integrated stress response activation and PKR signaling converge on eIF2α phosphorylation, thereby shifting translation toward stress-adaptive programs while suppressing cap-dependent protein synthesis [115]. This creates both a challenge and an opportunity for therapeutic mRNA design: while immune activation can reduce overall protein yield, stress-adaptive regulatory elements (e.g., IRES-like mechanisms, uORF reprogramming, and RNA structure-dependent initiation) may retain partial translational activity under these conditions [116].

Finally, manufacturing quality control is directly linked to these biological effects, as variations in RNA purity, capping efficiency, and poly-(A) length distribution can alter the degree of innate immune activation and thus indirectly influence the effectiveness of stress-responsive translational strategies [117]. A more integrated view of therapeutic mRNA performance should therefore consider RNA engineering, innate immune sensing, and manufacturing consistency as interconnected determinants of translational output in stressed cellular environments.

## 8. Conclusions

Balancing multiple design parameters during therapeutic mRNA optimization remains one of the major challenges in the development of RNA-based medicines. Although recent advances in deep-learning-assisted UTR engineering and stress-responsive mRNA design have significantly expanded the possibilities for translational control [118,119], the predictive performance of engineered constructs in physiologically relevant in vivo environments remains limited. Cis-regulatory elements influence not only translation efficiency, but also RNA stability, intracellular trafficking, interactions with RNA-binding proteins and endogenous non-coding RNAs, and activation of alternative translation pathways that are highly dependent on cellular context [120,121,122] Consequently, universal design solutions applicable across different tissues, disease states, and stress conditions are unlikely to be feasible.

Despite these limitations, stress-adaptive mRNA design remains a promising strategy for improving therapeutic selectivity and expression in diseased tissues. Several approaches, including stress-responsive UTR selection, nucleoside modifications, and alternative translation mechanisms, have demonstrated encouraging preclinical activity. For instance, 2′-*O*-methylation within the 5′ cap improves mRNA expression under IFN-α-associated stress conditions [123], whereas pseudouridine incorporation enhances RNA stability while reducing innate immune activation [124]. Similarly, combining alternative translation-supporting 5′UTRs with modified cap structures has been shown to enhance translation in stress-adapted cellular contexts [125] and tissue-selective UTRs such as the Ces1d-derived 5′UTR improved mRNA expression in the post-myocardial infarction heart [126].

Future progress in this field will require integrated evaluation of translational efficiency, delivery biology, stress signaling, and safety profiling in physiologically relevant in vivo systems. Combining ribosome profiling, RNA structural modeling, stress mapping, and systems-level computational analyses may enable the development of adaptive mRNA therapeutics that achieve context-selective expression while minimizing interference with endogenous stress responses and off-target translational activation.

## Figures and Tables

**Table 1:** Stress-responsive translational regulatory mechanisms and their current validation status in therapeutic mRNA engineering.

Regulatory Mechanism	Biological Role in Endogenous mRNAs	Proposed Application in Therapeutic mRNA Design	Validation Status in Engineered Therapeutic mRNAs
Upstream open reading frames (uORFs)	Translation initiation during stress, often through ISR/eIF2α signaling	Conditional control of translation under stress conditions	Mostly demonstrated in endogenous systems; limited validation in synthetic therapeutic mRNAs [93]
Internal ribosome entry sites (IRESs)	Enable cap-independent translation in cellular stress	Sustaining translation when cap-dependent initiation is inhibited	Experimentally validated in several engineered mRNA systems [94]
Cap-independent translation enhancer (CITE) elements	Promote translation independently of canonical cap recognition	Enhancing translation efficiency in stress-exposed cells	Primarily experimental and preclinical evidence [95]
TISU motifs	Support efficient translation initiation under energy stress and low ATP conditions	Maintaining translation in metabolically stressed tissues	Limited but promising validation in vitro and in vivo [96]
m^6^A RNA modification	Regulates mRNA stability, localization, and stress-responsive translation	Fine-tuning translation and stress adaptation of therapeutic mRNAs	Increasing experimental support in engineered mRNAs [97,98]
microRNA-responsive elements	Control mRNA stability and translation in a cell-specific manner	Tissue-selective or stress-dependent regulation of therapeutic expression	Widely used in experimental therapeutic mRNA platforms [99,100]
RNA-binding protein (RBP)-mediated regulation	Modulates mRNA localization, stability, and translation during stress	Engineering stress-responsive translational control elements	Mainly supported by in vitro studies [98,101]

## Data Availability

The data presented in this study are openly available in Zenodo [10.5281/zenodo.19701247].

## Abbreviations

The following abbreviations are used in this manuscript:

mRNA	Messenger RNA
5′UTR	5′ Untranslated Region
3′UTR	3′ Untranslated Region
tRNA	Transfer RNA
43S PIC	43S Pre-initiation Complex
eIF	Eukaryotic Translation Initiation Factor
GEF	Guanine Nucleotide Exchange Factor
eIF2–GDP	Eukaryotic Initiation Factor 2–Guanosine Diphosphate
eIF2–GTP	Eukaryotic Initiation Factor 2–Guanosine Triphosphate
eIF4	Eukaryotic Translation Initiation Factor 4
eEF2K	Eukaryotic Elongation Factor 2 Kinase
TC	Ternary Complex
GTP	Guanosine triphosphate
ISR	Integrated Stress Response
GCN2	General Control Non-derepressible-2
PERK	PKR-like ER Kinase
PKR	Protein Kinase RNA
HRI	Heme-regulated Inhibitor Kinase
uORFs	Upstream Open Reading Frames
ATF4	Activating Transcription Factor 4
IRESs	Internal Ribosome Entry Sites
BiP	Binding Immunoglobulin Protein
APAF-1	Apoptotic Protease Activating Factor-1
RBPs	RNA-binding Protein
HuR	Human Antigen R
TIA-1	T-cell intracellular antigen-1
mTOR	Mammalian Target of Rapamycin
4E-BPs	eIF4E-binding Proteins
PI3K	Class I Phosphoinositide 3-Kinase
AMPK	5′-Adenosine Monophosphate-activated Protein Kinase
5′TOP	5′Terminal Oligopyrimidine
CCND1	Cyclin D1
VEGFA	Vascular Endothelial Growth Factor A
ODC1	Ornithine Decarboxylase
MNKs	Mitogen-activated Protein Kinase (MAPK)-interacting Kinases
TSC	Hamartin
MDM2	Mouse Double Minute 2
BAX	Bcl-2-associated X Protein
AGO2	Argonaute 2
FXR1	Fragile X-related Protein 1
tRFs	tRNA-derived RNA Fragments
tiRNAs	tRNA-derived Stress-induced RNAs
ANG	Angiogenin
YTHDF1	YTH N6-Methyladenosine RNA-binding Protein 1
CHOP	DNA Damage-inducible Transcript 3
ATG5	Autophagy Related 5
PABP	Poly(A)-binding Protein
TISU	Translation Initiator of Short 5′ UTR
TSS	Transcription Start Site
IRES	Internal Ribosome Entry Site
VEGF	Vascular Endothelial Growth
HIF-1α	Hypoxia-inducible Factor 1-alpha
CITE	Cap-independent Translation Enhancers
